# The Role of Brain-Derived Neurotrophic Factor in Obstructive Sleep Apnea and Endothelial Function in a Pediatric Population With Obesity

**DOI:** 10.3389/fmed.2021.835515

**Published:** 2022-01-20

**Authors:** Sanae Makhout, Eline Vermeiren, Karolien Van De Maele, Luc Bruyndonckx, Benedicte Y. De Winter, Kim Van Hoorenbeeck, Stijn L. Verhulst, Annelies Van Eyck

**Affiliations:** ^1^Laboratory of Experimental Medicine and Pediatrics and Member of the Infla-Med Centre of Excellence, University of Antwerp, Antwerp, Belgium; ^2^Department of Pediatrics, Antwerp University Hospital, Edegem, Belgium; ^3^Department of Pediatric Cardiology, Amsterdam University Medical Centers, Amsterdam, Netherlands; ^4^Department of Gastroenterology and Hepatology, Antwerp University Hospital, Edegem, Belgium

**Keywords:** brain-derived neurotrophic factor, pediatric obesity, obstructive sleep apnea, endothelial function, weight loss treatment, multidisciplinary obesity treatment

## Abstract

**Background:**

Childhood obesity has increased worldwide, becoming a significant public health concern. Brain-derived neurotrophic factor (BDNF) plays an important role in the central regulation of food intake and body weight, but little is known regarding its role in childhood obesity. Next to obesity, BDNF has been linked to obstructive sleep apnea (OSA) and endothelial dysfunction, two obesity-related comorbidities. The aim of this study is to investigate how BDNF, OSA and endothelial dysfunction interact in children with obesity and to determine the effect of weight loss on serum BDNF levels.

**Methods:**

Children and adolescents with obesity aged 8–18 years who were enrolled in a multidisciplinary obesity treatment (MOT) in a tertiary hospital, were prospectively included. Several examinations were conducted during this MOT; at baseline, after 6 months and after 12 months, including the assessment of endothelial function, body composition measurements and a polysomnography. BDNF levels were measured on a serum sample by means of ELISA.

**Results:**

A total of 103 patients with obesity was included, of which 20 had OSA (19.4%). BDNF levels were comparable in children with obesity and OSA and children with obesity but without OSA (26.75 vs. 27.87 ng/ml, *p* = 0.6). No correlations were found between BDNF and sleep-related variables or between BDNF and endothelial function parameters nor between BDNF and adiposity measures. To investigate if the interaction between OSA and endothelial dysfunction had an influence on BDNF levels, a general linear model was used. This model revealed that a diagnosis of OSA, as well as the interaction between OSA and maximal endothelial dilatation, contributed significantly (*p* = 0.03, *p* = 0.04, respectively) to BDNF levels. After 1 year of weight loss therapy, BDNF levels did not change (26.18 vs. 25.46 ng/ml, *p* = 0.7) in our population.

**Conclusion:**

BDNF concentrations were comparable in children with obesity, both with and without OSA, indicating that BDNF levels are not affected by OSA. However, we did find an interaction effect of OSA and endothelial function on BDNF levels.

## Introduction

The pediatric obesity epidemic has been expanding in both developed and developing countries at an alarming rate over the past decades. According to the World Health Organization, 340 million children and adolescents worldwide had overweight or obesity in 2016 (1). Multiple cardiovascular risk factors are associated with childhood obesity, which can all detrimentally affect the endothelium, leading to endothelial dysfunction (2–4), highly prognostic of later cardiovascular morbidity and mortality. Another important obesity-related comorbidity is obstructive sleep apnea (OSA) which is characterized by recurrent breathing pauses during sleep. Several complications are associated with OSA in childhood, including cardiovascular and neurocognitive complications (5, 6). Studies show that the recurrent episodes of arousal and hypoxemia, occurring in OSA patients, can result in increased sympathetic activity, oxidative stress and inflammation, again contributing to endothelial dysfunction (7, 8). This implies that inflammatory processes leading to endothelial dysfunction can play an important role in the association between OSA and cardiovascular disease. Since obesity is a leading cause of OSA in adults and an increasing etiology of childhood OSA, there is concern that the two concurrent conditions may create an environment that further induces endothelial dysfunction and cardiovascular morbidity (9). This was confirmed by a study that showed that both OSA and obesity are independently associated with an increased risk for endothelial dysfunction in prepubertal children, and that these effects are magnified when both obesity and OSA are present (10). Indeed, our research group recently showed that even after weight loss treatment endothelial dysfunction improved less in the presence of OSA (11). This highlights the need for effective long-term weight-loss programs to prevent OSA and the ongoing epidemic of obesity.

Brain-derived neurotrophic factor (BDNF) is one of the neurotrophic factors that support differentiation, maturation and survival of neurons in the nervous system. Besides its neuroprotective effect, BDNF plays a major role in feeding behavior, food intake regulation, energy metabolism and weight control (12, 13). However, the association between circulating levels of BDNF and obesity is still unclear. Only a limited number of studies have looked at the relationship between BDNF and obesity in children, with conflicting results (14, 15). No studies have investigated the effect of OSA and obesity on BDNF in a pediatric population. Animal studies have shown that exposure to chronic intermittent hypoxia can lead to a decrease in BDNF expression (16). As BDNF has an important role in cognitive function (17), this could indicate a crucial role for this protein in the neurocognitive complications seen in childhood OSA. Since it has been suggested that neurocognitive deficits and endothelial dysfunction as seen in children with OSA are linked (18), BDNF could play an important role in this association (19). Indeed, numerous reports have uncovered critical roles for neurotrophins such as BDNF and their receptors on non-neural cells, including endothelial cells (20). Therefore, this is the first study that examines the relationship between OSA, endothelial function and BDNF in a pediatric population with obesity. Additionally, the effect of a multidisciplinary obesity weight loss treatment program on BDNF levels in this population is also investigated.

## Materials and Methods

### Study Design

In this prospective study, children and adolescents with obesity aged 8–18 years were consecutively included through the pediatric obesity clinic of the Antwerp University Hospital. Patients were excluded in case of an acute inflammatory process; use of non-steroidal anti-inflammatory drugs or immunosuppressive drugs; structural heart disease or other cardiac diseases; active malignant hematological disease; an underlying syndromic disorder; and a genetic or endogenous cause of obesity. Part of the study population was included via a randomized controlled trial (n°ISRCTN14722584) of which the results were previously reported (21, 22).

All patients were asked to complete three study visits: a baseline visit at the moment of inclusion, a second visit after 6 months of treatment (Follow-up 1) and a third visit after 1 year of weight loss treatment (Follow-up 2).

The ethics committee of the Antwerp University Hospital approved this study (EC no. 19/45/519 and 17/10/112) and a written informed consent was obtained from patients and their parents or legal guardian.

### Multidisciplinary Obesity Treatment

Each participant in this study received a treatment program based on an evidence-based multidisciplinary obesity treatment protocol (MOT) at the pediatric obesity clinic of the Antwerp University Hospital. The multidisciplinary team consists of an experienced pediatrician and pediatric dietician. Personal psychological support is provided on request. Briefly, the program consists of a first contact after which a 2-day inpatient stay in the hospital is planned. During this hospitalization the severity of obesity and its associated co-morbidities (such as cardiovascular disease, OSA, insulin resistance and liver steatosis) are further objectified. Results are discussed with patients and their parents. After this, a routine follow-up is established where the dietician is seen on a 4 weekly basis and works on a step-by-step approach to establish a sustainable healthy food pattern. The pediatrician is seen on a 3-monthly basis and monitors the evolution of the obesity severity and its related co-morbidities. Throughout the sessions, physical activity with a minimum of 30 min a day is highly encouraged.

### Anthropometry

Height was accurately measured to 0.1 cm using a standing stadiometer and weight was measured to the nearest 0.05 kg using a digital weighing scale. Waist circumference and waist-to-hip ratio were measured by standardized techniques, using an inelastic, retractable tape measuring the smallest circumference between the lowest ribs and highest hip comb ~1 cm above the umbilicus. Body mass index (BMI) was calculated as weight in kilograms over height in m^2^ and was further analyzed as *z*-scores using the Flemish growth study as a reference population (23). Overweight and obesity were defined according to the International Obesity Task Force criteria (24).

### Blood Pressure

The arterial blood pressure was each visit measured by an automated validated oscillometric device at the right upper arm with an adjusted cuff. The blood pressure was measured three times and an average of these measurements was used. Systolic and diastolic blood pressure percentiles were calculated specified for age, height and sex (25, 26).

### Body Composition

The body composition was measured by a body composition monitor® (Fresenius Medical Care, St. Wendel, Germany) relying on the technique of bioimpedance spectroscopy. Each measurement was performed in the morning after an overnight fast, with the patient lying supine. Electrodes were attached following the wrist-ankle approach, in a tetrapolar arrangement with two electrodes placed on the hands and two on the feet. To guarantee good contact of the electrodes with the skin, degreasing with diethylether was performed before placement of the electrodes. Age, sex, height, weight and blood pressure were registered by the device before starting the measurement. If the quality calculated by the BCM was below 75%, the measurement was repeated and only good quality measurements were used (27). All guidelines for the use of the BCM were as follows: non-electrical bed, no cell phones and no electrical devices within 1 m of the device.

### Endothelial Function

Peripheral microvascular endothelial function was non-invasively measured at the distal phalanx of the index finger using the Endo-PAT 2000 (Itamar Medical Ltd., Israel). Measurements were performed in the morning after an overnight fast in a temperature-controlled room (21–24°C). Briefly, finger probes were placed at the fingertips of both hands to measure arterial pulse wave amplitudes. After a 5-min baseline measurement, the brachial artery in the non-dominant arm was occluded using a manometer cuff to supra-systolic pressures (≥200 mmHg). After 5 min of occlusion, the cuff was released to allow recording of the reactive hyperemia for 5 min. Measurements were performed according to manufacturers' guidelines and recommendations in children (28). Parameters of interest are reactive hyperemia index (RHI), mean baseline pulse wave amplitude (PWA), maximal dilation and time to peak response.

### Sleep Assessment

At baseline, all children underwent standard nocturnal polysomnography evaluation in the child sleep laboratory of the Antwerp University Hospital with assessment of following variables that were continuously measured and recorded by a computerized polysomnograph (Brain RT, OSG, Rumst, Belgium): electroencephalography (C4/Al and C3/A2); electro-oculography; electromyography of anterior tibialis and chin muscles; and electrocardiography. Respiratory effort was measured by respiratory inductance plethysmography and oxygen saturation by a finger probe connected to a pulse oximeter (Xpod, Nonin, Minnesota, USA). Airflow was measured by means of a nasal pressure cannula and thermistor, and snoring was detected by means of a microphone at suprasternal notch. Children were also monitored on audio/videotape using an infrared camera (29, 30).

An apnea was defined as the absence of airflow lasting at least two breaths, a hypopnea as a ≥30% decrease in amplitude of the airflow signal, lasting for ≥2 breaths and with a concurrent desaturation of ≥3% or a concurrent arousal. An obstructive apnea was diagnosed in the presence of continued or increased respiratory effort. In the absence of respiratory effort, the apnea was labeled as a central apnea. The number of obstructive apneas and hypopneas per hour of sleep were quantified in the obstructive apnea-hypopnea index (oAHI). OSA is classified as mild (oAHI 2–5/h), moderate (>5 and up to 10/h) and severe (oAHI > 10 events/h) (29, 30). All desaturations of ≥3% from the baseline SaO_2_ were quantified and the oxygen desaturation index was calculated as the total of desaturations divided by the total sleep time (31, 32).

Weight loss was seen as the initial treatment for OSA, and therefore no extra treatment was given between study visits. Severe cases of OSA were referred to an ENT specialist for further evaluation.

### Determination of Human Free Serum BDNF Concentrations

A fasting venous blood sample was drawn to determine BDNF levels using a sandwich Enzyme-Linked Immunosorbent Assay (ELISA) (Quantikine; R&D Systems, Inc., Minneapolis, MN), according to the manufacturers' guidelines. The detection limit of the assay was 20 pg/ml, the intra-assay coefficient of variation was 13.85% and the intra-assay coefficient of variation was <10% for all samples.

### Statistical Analysis

All statistical analysis was performed using the Statistical Package for Social Sciences (SPSS, version 27, NY, USA). Normality was tested using a Kolmogorov–Smirnov test. Normally distributed data were presented as mean and standard deviation and skewed data were reported as median and range (minimum–maximum). Patients were distributed in groups based on their oAHI. Groups with OSA and groups without OSA were compared using the independent samples *T*-test for normally distributed data and the Mann-Whitney U test for skewed data. To look for correlations between parameters, the Pearson correlation was used for normally distributed data and the Spearman correlation for skewed data. Afterwards, general linear models were used to investigate if the interaction between OSA and endothelial function had an effect on BDNF levels. For all analyses, *p* < 0.05 was considered statistically significant. Based on a previous study that found a significant correlation between oxygen desaturation index (ODI) and BDNF (*r* = 0.65, *p* < 0.001) (33), a sample size was calculated that would need 16 subjects with OSA to achieve adequate statistical power (power goal of 80% and a type I error rate of 5%).

## Results

### Baseline Assessment

**Figure 1:**
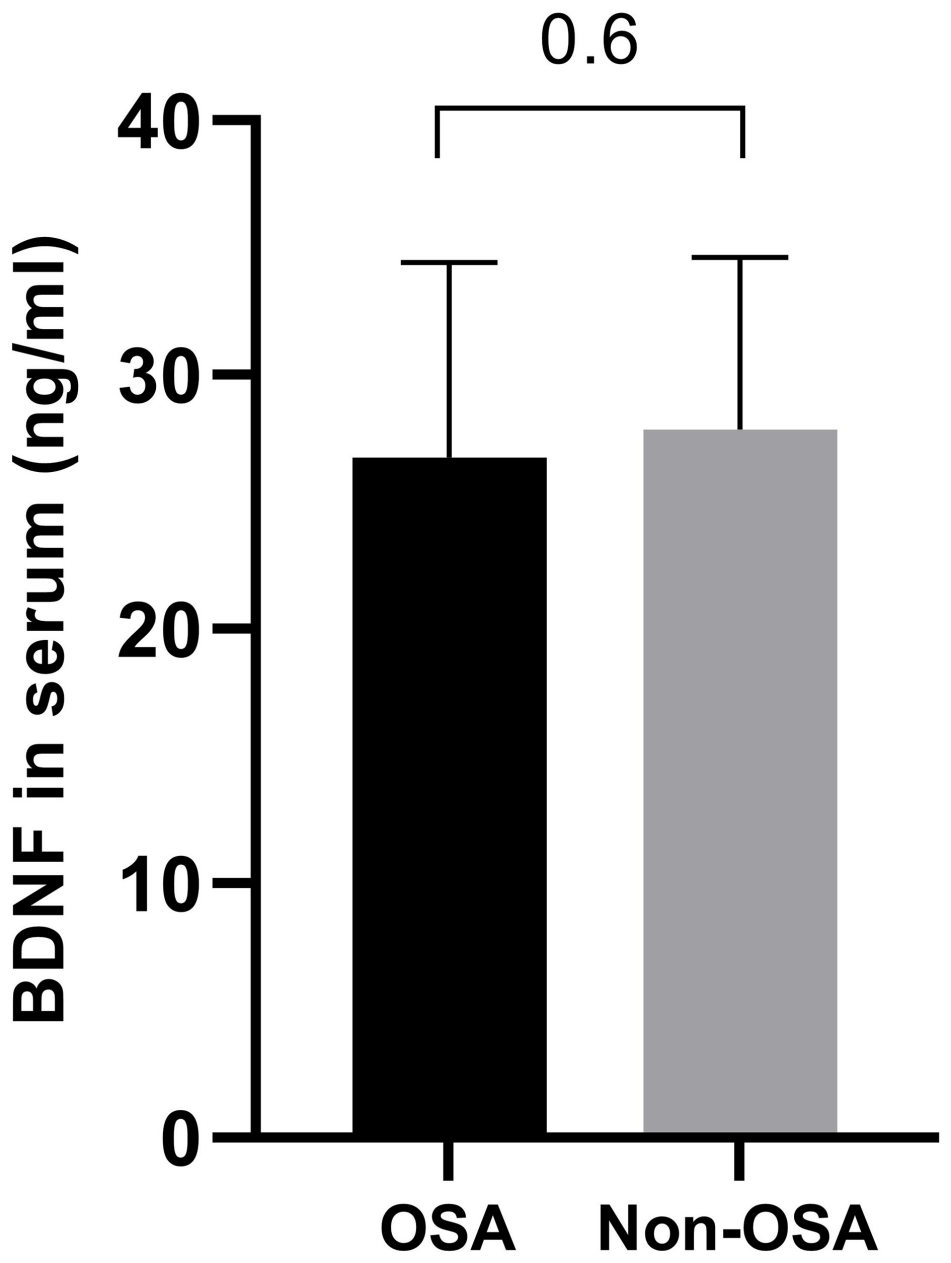
Comparison of BDNF levels between patients with (oAHI ≥ 2) and without OSA (oAHI <2). The BDNF level is comparable in both groups (26.75 ± 7.66 ng/ml vs. 27.87 ± 6.77 ng/ml, *p* = 0.6).

A total of 103 children with overweight or obesity were included with an average BMI of 31.14 kg/m^2^ (range 21.89–40.89 kg/m^2^) corresponding to a mean *z*-score of 2.45 (range 1.70–3.20). The mean age was 12.02 ± 2.17 years, and 51.5% of subjects were female. OSA was detected in 20 patients (19.4%): 12 children had mild OSA (60%) and 8 children had moderate-to-severe OSA (20%). Characteristics of obese patients with (oAHI ≥ 2) and without (oAHI <2) OSA are presented in Table 1. Both groups were comparable in age and body composition. In our population, more boys were diagnosed with OSA (*p* = 0.03) and the diastolic blood pressure percentile (*p* = 0.03) was higher in patients with OSA. As expected, several sleep-related variables were significantly higher in the OSA group. Table 2 shows that the mean baseline PWA (416.60 vs. 411.51, *p* = 0.002) was significantly higher in the OSA group. No significant differences were found in BDNF levels between subjects with and without OSA (Figure 1).

**Table 1:** Characteristics and sleep assessment data of patients with (oAHI ≥ 2) and without (oAHI <2) obstructive sleep apnea at baseline.

	**OSA (oAHI ≥2)**	**Non-OSA (oAHI <2)**	***p*-value**
*N*	20	83	
Sex (male/female)	14/6	36/47	0.03
Age (years)	12.8 ± 2.6	12.3 ± 2.1	0.4
Weight (kg)	84.33 ± 19.58	78.92 ± 19.99	0.3
Height (cm)	159.35 ± 11.85	158.49 ± 12.18	0.8
BMI (kg/m^2^)	32.76 ± 4.06	30.74 ± 4.18	0.06
BMI *z* score	2.50 (1.90–3.20)	2.40 (1.70–3.10)	0.1
Waist (cm)	95.8 (73.5–117.0)	91.0 (65.0–118.0)	0.1
WHR	0.90 (0.73–1.00)	0.87 (0.74–1.06)	0.1
Systolic blood pressure (mmHg)	116.20 ± 11.76	111.14 ± 11.02	0.07
Diastolic blood pressure (mmHg)	69 (58–87)	63 (46–88)	0.04
Systolic blood pressure percentile	79 (6–99)	66 (3–99)	0.08
Diastolic blood pressure percentile	72 (21–98)	46 (4–99)	0.03
Lean % (%)	45.59 ± 5.58	47.56 ± 6.40	0.5
Fat % (%)	41.71 ± 4.15	40.49 ± 4.44	0.3
oAHI (events/h)	3.15 (2.00–14.40)	0.48 (0.00–1.70)	<0.001
ODI (events/h)	1.65 (0.00–6.90)	0.20 (0.00–5.10)	<0.001
TST 95 (%)	0.10 (0.00–2.60)	0.00 (0.00–3.90)	0.003
Mean SaO_2_ (%)	96.84 ± 0.77	97.08 ± 0.74	0.2
Number of desaturations	55 (1–268)	11 (0–136)	<0.001

*BMI, body mass index; BMI z, BMI standard deviation; WHR, waist-to-hip ratio; oAHI, obstructive apnea-hypopnea index; ODI, oxygen desaturation index; TST, total sleep time; TST 95, total sleep time with saturation under 95%; mean SaO_2_, mean oxygen saturation*.

**Table 2:** Endothelial function parameters of patients with (oAHI ≥ 2) and without (oAHI <2) obstructive sleep apnea at baseline.

	**OSA (oAHI ≥2)**	**Non-OSA (oAHI <2)**	***p*-value**
RHI	1.37 (0.92–2.45)	1.59 (0.80–3.03)	0.1
Mean baseline PWA	416.60 ± 200.11	411.51 ± 192.26	0.002
TPR (seconds)	202 (75–285)	195 (45–285)	0.7
Maximal dilation	1.49 (1.12–2.64)	1.29 (0.84–2.47)	0.2

*RHI, reactive hyperemia index; mean baseline PWA, mean baseline pulse wave amplitude; TPR, time to peak response*.

To investigate the impact of OSA and endothelial dysfunction on BDNF levels, a correlation analysis was performed. No correlations were found between BDNF and any of the sleep-related variables (*p* > 0.2), nor between BDNF and endothelial function (*p* > 0.1). Furthermore, BDNF was not associated with any obesity-related parameter or patient characteristics. To investigate whether the interaction between OSA and endothelial function had an effect on BDNF levels, a general linear model with OSA diagnosis as a categorical variable and maximal dilatation was made. This model (Table 3) shows that a diagnosis of OSA (*p* = 0.03) and the interaction between OSA and maximal endothelial dilatation (*p* = 0.04) both contributed significantly to BDNF levels. The maximal dilatation and BMI *z* score separately did not have an effect on BDNF levels (both *p* = 0.1).

**Table 3:** General linear model for BDNF with OSA diagnosis, BMI *z* score and maximal dilatation as factors.

	***r* ^2^**	***p*-value**
**BDNF**	0.07	
OSA diagnosis		0.03
BMI *z* score		0.1
Maximal dilatation		0.1
OSA diagnosis ^*^ maximal dilatation		0.04

*BDNF, brain-derived neurotrophic factor; OSA, obstructive sleep apnea*;

* *indicates an interaction effect*.

### Follow-Up Assessment

Of the 103 patients that participated at baseline, 82 (79%) attended the first follow-up visit and 73 patients (71%) attended the second follow-up visit. The average interval between baseline and follow-up visit 1 was 8.00 ± 1.18 months and between baseline and follow-up visit 2 was 15.00 ± 1.54 months. Patient characteristics at the different study visits are shown in Table 4. The mean BMI *z*-score at the start of treatment was 2.43 ± 0.36 and decreased significantly to 2.25 ± 0.43 at the first follow-up visit and to 2.22 ± 0.49 at the second follow-up visit (*p* <0.001). The mean absolute decrease in BMI *z* score after 1 year was 0.21 ± 0.14.

**Table 4:** Patient characteristics and body composition measurements of baseline and follow-up visits.

	**Baseline**	**Follow-up visit 1**	**Follow-up visit 2**	***p*-value**
N	103	82	73	
Sex (male/female)	50/53	40/42	38/35	
Age (years)	12.41 ± 2.21	13.00 ± 2.22	13.49 ± 2.35	<0.001^*^^#^^°^
Weight (kg)	80.54 ± 20.63	79.90 ± 20.90	83.07 ± 20.67	0.004^*^^#^^°^
Height (cm)	158.66 ± 12.06	161.29 ± 11.87	163.93 ± 11.48	<0.001^*^^#^^°^
BMI (kg/m^2^)	31.45 ± 4.73	30.25 ± 5.24	30.53 ± 5.46	<0.001^*^^#^^°^
BMI *z* score	2.40 (1.10–3.30)	2.30 (1.40–3.70)	2.29 (0.96–3.70)	<0.001^*^^#^
Waist (cm)	92.00 (65.00–128.50)	87.00 (68.00–125.00)	87.05 (67.50–118.00)	<0.001^*^^#^
WHR	0.87 ± 0.06	0.84 ± 0.07	0.83 ± 0.07	<0.001^*^^#^
Systolic blood pressure (mmHg)	112.13 ± 11.29	116.91 ± 14.97	117.01 ± 13.71	<0.001^*^^#^
Diastolic blood pressure (mmHg)	63.00 (46.00–88.00)	72.00 (51.00–100.00)	72.00 (53.00–95.00)	<0.001^*^^#^
Systolic blood pressure percentile	72 (3–99)	80 (8–99)	73 (1–99)	0.8
Diastolic blood pressure percentile	50 (4–99)	77 (14–99)	73 (11–99)	<0.001^*^^#^
Lean % (%)	46.80 ± 6.73	47.59 ± 8.01	46.47 ± 8.52	0.9
Fat % (%)	40.69 ± 5.28	39.70 ± 6.29	40.55 ± 6.72	0.4

*BMI, body mass index; BMI z, BMI standard deviation; WHR, waist-to-hip ratio*.

* *Significant difference between baseline and follow-up visit 2*.

# *Significant difference between baseline and follow-up visit 1*.

° *significant difference between follow-up visit 1 and follow-up visit 2*.

**Figure 2:**
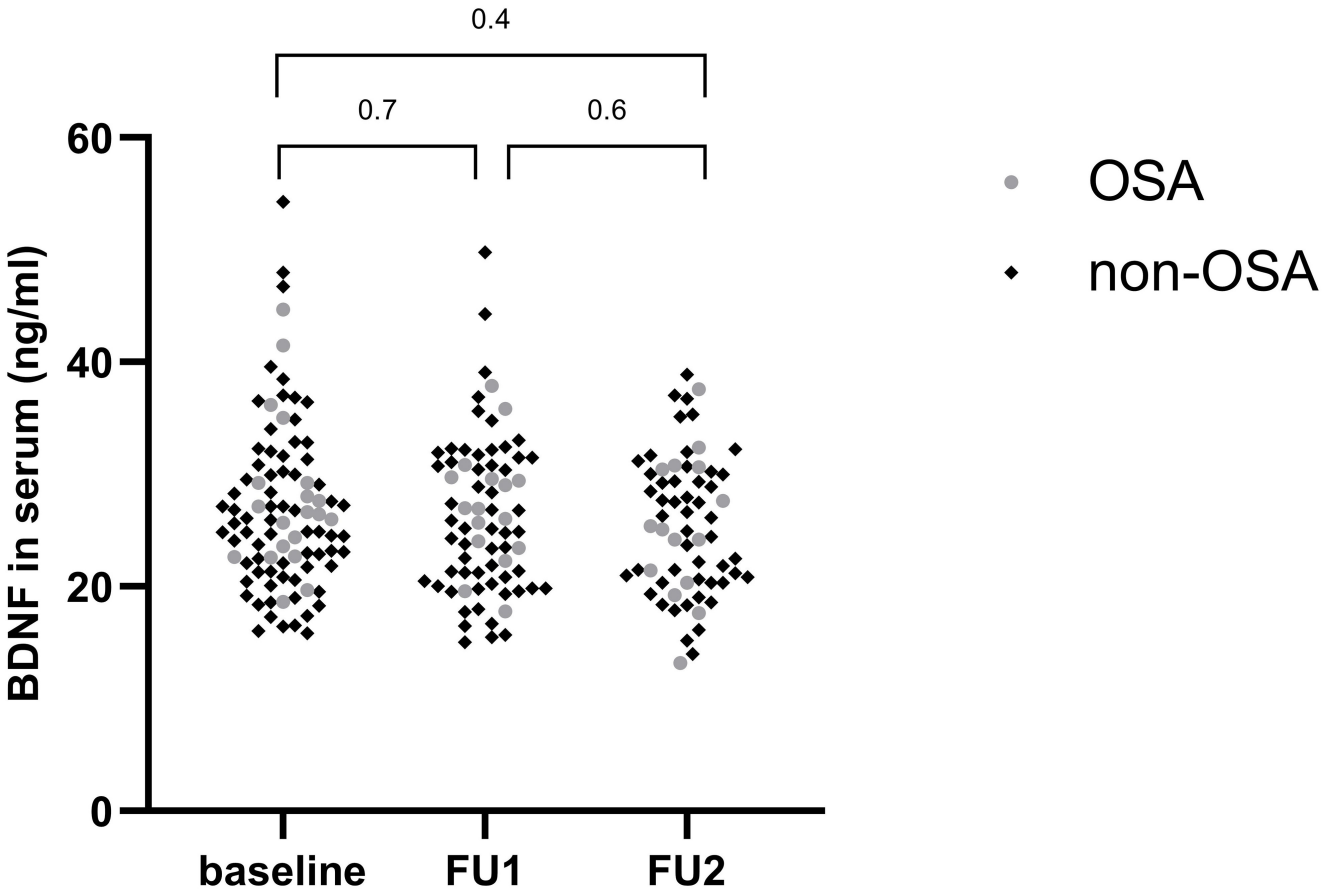
Evolution of BDNF levels during weight loss. FU1, Follow-up visit 1; FU2, follow-up visit 2.

Anthropometric data and their evolution in time are shown in Table 4. BDNF levels remained stable during weight loss (26.18 ng/ml at baseline vs. 25.46 ng/ml after 1 year, *p* = 0.4) (Figure 2). Changes in body weight (BMI *z* score) and changes in BDNF levels (BDNF) between baseline and after 1 year did not correlate (*r* = 0.22, *p* = 0.09), also no relationship was found between BMI *z* score and final BDNF levels measured after 1 year (*r* = −0.14, *p* = 0.27). The endothelial function parameters were not significantly different between baseline and the follow-up visits (Table 5), except for the maximum dilatation that significantly decreased between baseline and the first follow-up visit (1.52 vs. 1.47, *p* <0.001) and remained stable between the first and second follow-up visit (1.47 and 1.46, *p* = 0.1). Also, no correlations were found between (BDNF) and differences in maximal dilatation after 1 year (*r* = 0.007, *p* = 0.9) nor between maximal dilatation and final BDNF levels measured after one year (*r* = −0.09, *p* = 0.7).

**Table 5:** Endothelial function parameters during weight loss treatment.

	**Baseline**	**Follow-up visit 1**	**Follow-up visit 2**	***p*-value**
N	103	82	73	
RHI	1.52 (0.80–2.87)	1.47 (0.95–4.47)	1.48 (1.00–3.00)	0.5
Mean baseline PWA	443.73 ± 207.50	413.31 ± 207.23	426.35 ± 193.485	0.4
TPR (seconds)	195 (45–285)	165 (75–285)	165 (45–285)	0.4
Maximal dilation	1.52 (0.80–3.03)	1.47 (0.95–4.47)	1.46 (1.00–3.00)	<0.001^#^^*^

*RHI, reactive hyperemia index; mean baseline PWA, mean baseline pulse wave amplitude; TPR, time to peak response*.

# *Significant difference between baseline and follow-up visit 1*.

* *Significant difference between baseline and follow-up visit 2*.

## Discussion

In this prospective study, no difference in BDNF levels could be found between children with obesity, both with and without OSA. However, an interaction of endothelial function and OSA on BDNF levels was found in this pediatric population with obesity. This interaction suggests that BDNF levels decrease in the context of endothelial dysfunction and OSA. Furthermore, 1 year of weight loss therapy did not have an effect on BDNF levels in this clinical cohort.

We could not detect a difference in serum BDNF levels between obese children with and without OSA. Limited studies in children have investigated the relationship between BDNF and OSA and to the best of our knowledge, we are the first to investigate BDNF levels in a solely obese pediatric population. The study by Wang et al. investigated BDNF levels in children with OSA and healthy volunteers (34). They found no difference in BDNF levels between the two groups, which is in agreement with our results. In this study, the authors also studied the effect of adenotonsillectomy on BDNF levels and found that BDNF levels decreased 3 months after adenotonsillectomy which was associated with the improvement in sleep-disordered breathing. This indicates an effect of OSA on BDNF levels. However, this decrease disappeared again 12-months post-adenotonsillectomy, as BDNF levels were then similar between control subjects and OSA patients. A number of studies also investigated the relationship between OSA and BDNF in adults. Flores et al. reported that adults with obesity and untreated OSA had significantly higher BDNF serum levels compared to adults with obesity but without OSA. This study also showed a positive correlation between the ODI and BDNF levels in adult OSA patients (33). As OSA in adults is often more severe compared to childhood OSA, this could indicate that patients with more pronounced intermittent hypoxia are more likely to have higher levels of circulating BDNF (33). This could be explained by the neuroprotective role of BDNF following hypoxic events, as studies have shown increased BDNF levels after acute ischemic stroke or repeated hypoxic stimulation in animal studies (35, 36). In this study, OSA was indeed more severe in their adult population than our pediatric population. In contrast, Staats et al. did not find any difference in BDNF levels at baseline between adult patients with untreated OSA and healthy controls. However, after CPAP treatment a decrease in BDNF levels was seen, again indicating an association between OSA and BNDF levels (37).

Children with obesity are known to exhibit impaired endothelial function (38). In our population, no difference in endothelial function between children with OSA and without OSA was found. Endothelial function did improve after weight loss in our population. However, the improvement of endothelial function did not have an effect on BDNF levels after 1 year of weight loss treatment.

At baseline, no correlations were found between BDNF and endothelial function parameters for our obese population or between BDNF and sleep-related variables. However, a general linear model for BDNF showed that the interaction between OSA and endothelial function contributed significantly to BDNF levels. Studies have shown that OSA and endothelial function are independently associated (39–42). Recently, our research group has also shown that OSA has an important influence on the improvement of endothelial function in a pediatric population with obesity (11). This study further demonstrates that the coexistence of OSA and endothelial function can have detrimental effects on the health of these children. BDNF levels are essential in the survival, growth and differentiation of new neurons during early brain development and are involved in the plasticity changes related to learning, memory, and higher cognitive function (17). Lower BDNF levels have also been linked with poorer neurocognitive function in children with type I diabetes (43). Our results indicate a possible role for BDNF in the neurocognitive complications seen in pediatric OSA. In the study by Gozal et al. a degree of concordance between endothelial function and the presence of cognitive deficits was found, suggesting a shared pathogenetic mechanism between endothelial dysfunction and neurocognitive deficits as both these disorders are more likely to coexist in children with OSA (18). It is possible that BDNF plays a role in the common pathophysiological pathways of neurocognition and endothelial function in pediatric OSA.

One year of weight loss treatment had no significant effect on BDNF levels over time in our study cohort. This result is comparable to a study by Lee et al., where BDNF levels remained stable after 12 weeks of weight loss. However, a significant increase in BDNF levels was found after the 12-week weight loss program (44). In contrast, Glud et al. showed that in both men and women a decrease in BDNF levels was caused after a 12-week weight-loss intervention (45). Since changes in body weight in humans have been shown to affect BDNF levels (46, 47), it is suggested that the effect of weight loss on circulating BDNF might depend on the level of exercise and dietary energy restriction (48). In contrast to a previous study in children with obesity where plasma concentrations of BDNF increased after weight loss and an adequate carbohydrate intake (15), we did not find a relationship between body weight changes and changes in BDNF levels after a weight-loss treatment of 1 year. The difference could be explained by the high variation in body weight change in our population. Also, in adults with overweight and obesity similar findings were found by Araya et al., as BDNF levels increased after a 3-month reduced-caloric diet, suggesting that BDNF levels can be regulated by food intake (47). It would be interesting to further investigate the potential dose-response relationship between BDNF levels and energy restriction and for the existence of a possible weight loss/energy restriction threshold.

This study has some strengths and limitations. The major strengths include the prospective design and the large study population with obesity. The measurement of BDNF in serum is an asset as previous studies have shown that measuring circulating BDNF in serum has several advantages compared to plasma or whole blood measurements (49, 50). Several study limitations need to be taken into consideration. First, the majority of our population consisted out of patients without OSA or with only mild OSA (7.8% of all patients were diagnosed with moderate to severe OSA). However, our population is representative of a pediatric obesity clinic since all patients with obesity were consecutively included and not only those suspected of sleep-disordered breathing. Second, we observed heterogeneous weight trajectories, limiting the average weight loss, as result that it was more difficult to detect a significant effect of weight than e.g., in a residential cohort. Third, as we did not assess cognitive function in our study, it was not possible to investigate the relationship between BDNF and cognitive function directly. Lastly, because of the COVID pandemic, appointments had to be rescheduled, which resulted in more variation in time between the different visits.

For future research it would be interesting to include a normal-weight control group to get a better understanding of the relationship between obesity and BDNF. Additionally, as polysomnography data was only available at baseline, it would be of interest to investigate the long-term association between OSA and BDNF and also include follow-up sleep data in future research.

To conclude, BDNF concentrations of children with obesity with and without OSA are comparable in our cohort, suggesting that BDNF levels are not affected by OSA. An univariate association between BDNF and sleep-related variables or between BDNF and endothelial function parameters could not be determined. However, a general linear model showed that the interaction between OSA and endothelial function affects BDNF levels, indicating an indirect link between BDNF, OSA and endothelial function. Lastly, weight loss did not have an effect on BDNF levels. Future research with emphasis on longitudinal studies is necessary to investigate the possible role of BDNF in OSA and endothelial function, as it is complicated in children with conflicting data in current literature.

## Data Availability Statement

The raw data supporting the conclusions of this article will be made available by the authors, without undue reservation.

## Ethics Statement

The studies involving human participants were reviewed and approved by the Ethics Committee of the Antwerp University Hospital. Written informed consent to participate in this study was provided by the participants' legal guardian/next of kin.

## Author Contributions

The current study was conceptualized by AV, SV, and KVH. AV, EV, SV, KVH, and BD designed the methodology. The formal analysis was done by SM. The experiments were performed by SM, EV, KVDM, and AV. The original draft was prepared by SM and AV. EV, KVDM, LB, BD, KVH, and SV reviewed the original version. AV supervised the whole research activity together with SV, KVH, and BD. The project administration was executed by AV. The funding for this research project was obtained by SV, LB, and BD. All authors contributed to the article and approved the submitted version.

## Funding

Funding was received by the Research Foundation – Flanders: FWO TBM project no. 150179 but this organization had no conflict of interest with the results, nor a role in study design, the collection, analysis and interpretation of the data, the writing of the report or the decision to submit the article for publication.

## Conflict of Interest

The authors declare that the research was conducted in the absence of any commercial or financial relationships that could be construed as a potential conflict of interest.

## Publisher's Note

All claims expressed in this article are solely those of the authors and do not necessarily represent those of their affiliated organizations, or those of the publisher, the editors and the reviewers. Any product that may be evaluated in this article, or claim that may be made by its manufacturer, is not guaranteed or endorsed by the publisher.

## Abbreviations

AHI: apnea-hypopnea index
BDNF: brain-derived neurotrophic factor
BMI: body mass index
BMI z: BMI standard deviation score
ELISA: Enzyme-Linked Immunosorbent Assay
MOT: multidisciplinary obesity treatment
OAI: obstructive apnea index
oAHI: obstructive apnea–hypopnea index
OSA: obstructive sleep apnea
ODI: oxygen desaturation index
PWA: pulse wave amplitude
PSG: polysomnography
RHI: reactive hyperemia index
SaO2: oxygen saturation
TPR: time to peak response
TST: total sleep time
TST 95: total sleep time with saturation under 95%
WHR: waist-to-hip ratio.
